# Artificial Intelligence and Deep Learning in Revolutionizing Brain Tumor Diagnosis and Treatment: A Narrative Review

**DOI:** 10.7759/cureus.66157

**Published:** 2024-08-05

**Authors:** Shobha Mandal, Subhadeep Chakraborty, Muhammad Ayaz Tariq, Kamran Ali, Zenia Elavia, Misbah Kamal Khan, Diana Baltodano Garcia, Sofia Ali, Jubran Al Hooti, Divyanshi Vijay Kumar

**Affiliations:** 1 Internal Medicine, Guthrie Robert Packer Hospital, Sayre, USA; 2 Electronics and Communication, Maulana Abul Kalam Azad University of Technology, West Bengal, IND; 3 Medicine and Surgery, Quaid-e-Azam Medical College, Bahawalpur, PAK; 4 Internal Medicine, United Medical and Dental College, Karachi, PAK; 5 Medical School, Dr. D. Y. Patil Medical College, Hospital & Research Centre, Pune, IND; 6 Internal Medicine, Peoples University of Medical and Health Sciences, Nawabshah, PAK; 7 Ophthalmology, Antenor Orrego Private University, Trujillo, PER; 8 Medical School, Peninsula Medical School, Plymouth, GBR; 9 Medicine, University College Dublin, Dublin, IRL; 10 Internal Medicine, Smt. Nathiba Hargovandas Lakhmichand Municipal Medical College, Ahmedabad, IND

**Keywords:** convolutional neural network, brain tumor, neurosurgery, artificial intelligence, deep learning

## Abstract

The emergence of artificial intelligence (AI) in the medical field holds promise in improving medical management, particularly in personalized strategies for the diagnosis and treatment of brain tumors. However, integrating AI into clinical practice has proven to be a challenge. Deep learning (DL) is very convenient for extracting relevant information from large amounts of data that has increased in medical history and imaging records, which shortens diagnosis time, that would otherwise overwhelm manual methods. In addition, DL aids in automated tumor segmentation, classification, and diagnosis. DL models such as the Brain Tumor Classification Model and the Inception-Resnet V2, or hybrid techniques that enhance these functions and combine DL networks with support vector machine and k-nearest neighbors, identify tumor phenotypes and brain metastases, allowing real-time decision-making and enhancing preoperative planning. AI algorithms and DL development facilitate radiological diagnostics such as computed tomography, positron emission tomography scans, and magnetic resonance imaging (MRI) by integrating two-dimensional and three-dimensional MRI using DenseNet and 3D convolutional neural network architectures, which enable precise tumor delineation. DL offers benefits in neuro-interventional procedures, and the shift toward computer-assisted interventions acknowledges the need for more accurate and efficient image analysis methods. Further research is needed to realize the potential impact of DL in improving these outcomes.

## Introduction and background

Brain tumors are complex pathologies that can lead to neurological disability and functional impairment. The rate of new cases is approximately 200,000 people per year [1]. In recent years, artificial intelligence (AI) has been applied in numerous biomedical fields, including in the identification, diagnosis, and therapy of brain tumors. However, there is a gap in the integration of AI and medical practice [2].

A vast amount of clinical data is produced by the ever-expanding, versatile, and dynamic field of medicine in the form of imaging, patient electronic medical records, and laboratory results. Precision and personalized treatment are impeded by the sheer volume of medical data, which makes it impossible for physicians to manually process and extract patient data and evaluate specific, pertinent patient information as needed [2]. Recent developments in digitalization and technology hold the potential to overcome this barrier. Deep learning (DL) is one such AI tool that helps mitigate barriers to processing and accessing medical data [3].

The 1956 Dartmouth Conference saw the discovery of AI. It sought to use machines to mimic human memory and learning. Advancements in AI algorithms allow for the retrieval and use of increased amounts of clinical data for training [3]. Without requiring physicians to handle the raw structured data directly, DL is an improved form of machine learning, a former part of AI, that can automatically process and extract multilayer information from a significant amount of raw clinical data [4].

In recent years, radiodiagnostics, which includes medical imaging such as computed tomography (CT), magnetic resonance imaging (MRI), positron emission tomography (PET), ultrasound, X-ray, and much more, has grown considerably in significance and importance. This has aided in the prompt diagnosis and treatment of illnesses [5]. Most of the medical image interpretation is performed by medical professionals such as radiologists and physicians. However, researchers and medical professionals have lately started to realize the necessity for computer-assisted interventions for medical image analysis due to wide variances in pathology, probable human expert fatigue, and interindividual variability in radioimaging interpretation. Recent developments in machine learning, particularly in DL, have significantly advanced the ability to recognize, categorize, and quantify patterns in medical imaging and help resolve this need [6].

The development of sophisticated high-tech central processing units and graphics processing units, the availability of massive amounts of data (known as big data), and the evolution of AI learning algorithms have resulted in the success of DL [7]. In a technical sense, DL may be thought of as an evolved form of traditional AI algorithms that construct networks with more than two layers [6]. Empirical research demonstrates that deep neural networks can find hierarchical feature representations, which allow for the derivation of higher-level features from lower-level ones [8]. DL has demonstrated efficacy in medical image analysis, including image segmentation, image registration, image fusion, image annotation, computer-aided diagnosis and prognosis, lesion/landmark detection, and microscopic imaging analysis, to mention a few [3,6]. This is due to its unique characteristic of learning hierarchical feature representations solely from data. Furthermore, DL has lessened the barrier that prevents non-machine learning specialists from using DL for their studies and applications, particularly in the field of medical picture analysis [3]. DL requires only a set of data with minor preprocessing, if necessary, and then discovers the informative representations in a self-taught manner [2]. In this review, we aimed to explore how AI and DL are revolutionizing brain tumor diagnosis and treatment.

## Review

Methodology

A comprehensive literature search was conducted in PubMed, EMBASE, and Medline databases using a combination of the keywords “deep learning,” “artificial intelligence,” “brain tumor,” and “neuro-intervention.” The inclusion criteria included any type of article that discussed AI or DL applications in brain tumors. The exclusion criteria were articles for which full text was not available, were not in English, or were gray literature. The focus of the searched literature was the applications, advantages, challenges, and limitations of AI and DL in radiological and neuro-interventional procedures in the management and treatment of brain cancers. Additional references were identified by a manual search of the articles retrieved in the first round of the search. The last literature search was performed on June 15, 2024.

Applications of deep learning in the field of brain oncology

Usually, when we talk about preoperative planning, we talk about a holistic approach in which the patient undergoes an MRI/CT scan, comes back to the doctor and discusses the lesion, and then takes the next step with a neurosurgeon, radiologist, oncologist, anesthesiologist, etc. All of this depends entirely on the radiologist’s ability to detect the lesion correctly, be it its size, shape, intensity, and class in which it is placed. Inaccurate classification of the brain tumor can result in major consequences and reduce patient survivability. However, DL approaches have recently become popular in developing automated systems capable of accurately diagnosing or segmenting brain tumors in less time. DL enables a pre-trained convolutional neural network (CNN) model for medical images specifically for classifying brain cancers, and various applications such as GoogleLEnet, VGGNets, Alexnet, and resNet-34 are made [9].

Recent contributions include the Brain Tumor Classification Model (BCM-CNN), based on an advanced three-dimensional (3D) model using the Enhanced Convolutional Neural Network (BCM-CNN). It has the following two submodules: (i) CNN, hyperparameter optimization using an adaptive dynamic sine-cosine fitness grey wolf optimizer algorithm (ADSCFGWO) followed by a trained model, and (ii) segmentation module, the experimental results, show that the BCM-CNN, as a classifier, achieved the best results due to the enhancement of CNN’s performance by CNN optimization of hyperparameters [9]. Another significant contribution is the training model built with Inception-Resent V2, which improves brain tumor diagnosis and preoperative planning as its output is in binary 0 or 1, where 0 means normal, and 1 means tumor [9].

Recent advancements have led to the development of hybrid techniques. Pre-trained AlexNet, GoogLeNet, ShuffleNet, and ResNet networks are employed to extract features from tumor regions and adjacent tissues. Although deep features are crucial in the identification process, some low-level data about tumors may be lost. As a result, a shallow network is made to learn low-level data. Deep and shallow features are blended to compensate for the loss of data. With the fused feature sets, support vector machine and k-nearest neighbor classifiers are trained [9]. A proposal for a DL-based automatic multimodal classification technique for categorizing different brain tumors has five essential phases [9].

Another study presented a genetic algorithm (GA)-CNN hybrid for detecting glioblastoma and other brain cancers [10]. An appropriate CNN architecture was chosen automatically with the help of the genetic algorithm in this method. The authors were able to correctly identify glioma, meningioma, and pituitary cancer in 90.9% of cases and 94.2% of cases overall [11].

Another critical application of DL in medicine is image segmentation. A study reported a technique for fusing two-dimensional (2D) and 3D MRI images [12]. They suggested utilizing DenseNet for classification and unique 3D CNN architectures for the segmentation of multimodal pictures. On the test set, the proposed method performed admirably, with an accuracy of 92% using DenseNet and 85% using the individualized 3D CNN models.

DL AI also has the potential to substantially improve intraoperative diagnostic processes that are essential to real-time decision-making. When a tissue sample needs to be assessed in real-time during surgery, conventional methods involve transporting the sample to a laboratory for processing. Skilled and experienced laboratory personnel are required for hematoxylin and eosin staining and preparation of the sample, in addition to a pathologist to interpret the histological images [13]. This process requires extensive time, labor, and resource management. Furthermore, this process usually takes up to 30 minutes, which poses significant risks to the patient [14]. In contrast, a novel method of using AI intraoperatively in neurosurgery has been developed by Hollon et al., which can predict brain tumor diagnosis in real-time under 150 seconds. It is a method of parallel workflow that combines stimulated Raman histology (SRH), label-free optical imaging, and deep CNNs. After being trained with 2.5 million SRH images, the CNN has demonstrated in a prospective clinical trial to have 94.6% accuracy. It matches the accuracy of conventional methods, which yielded 93.9% accuracy, is more time efficient, and reduces the risks of prolonging surgery [13].

Neuronavigation is another example of AI integration in neurosurgery [15]. It involves computer-based image processing that can generate a singular frame of a frameless stereotactic navigation system. This novel technique can predict the location of lesions and anatomical structures at risk during various types of operations. In addition, this allows the surgeons to rely less on navigational datasets collected through intraoperative imaging and instead use more accurate navigational data that also accounts for shifts of structures during the operation. The use of neuronavigation has the potential to reduce patient risk during operation and facilitate the shift to more minimally invasive surgeries [16].

Advantages of using deep learning in the field of brain oncology

DL has the potential to improve accuracy and efficiency. It has shown usefulness in differential diagnosis in cases of difficult comparison by MRI evaluation between glioblastoma multiforme and primary central nervous system lymphoma. McAvoy et al., using EfficientNetB4 architecture in the CNN framework, analyzed contrast-enhanced T1-weighted images in 320 patients with suspected glioblastoma multiforme or primary central nervous system lymphoma [17]. Furthermore, in the early diagnosis of brain metastases, where stereotactic radiosurgery requires strict knowledge of the number and location of the metastases, computer-assisted automated detection is relevant in these cases. Radiogenomics is a combination of imaging and genotype that allows AI 1p19q detection related to oligodendroglioma and *EGFR* mutation in glioblastoma multiforme [18]. AI has been shown, with a 2D and 3D texture analysis model of T1-weighted post-contrast sequences, to characterize the tumor phenotype according to whether it was metastases from lung cancer, melanoma, or breast cancer as it can show differences in local environments. It allows surgical treatment planning, enabling us to determine the lesion segmentation. Boaro et al. demonstrated an accuracy of 88.2% using a 3D CNN [19]. However, computer-aided design must be used in an appropriate setting as there may be overdiagnosis if sensitivity thresholds are too low. If it is high, small lesions may not be detected [20].

One of the most significant risks to patients is excess radiation exposure, especially those with multiple conditions that require regular radiological check-ups. Excess radiation exposure can lead to an increased risk of cancer, and, therefore, reducing this risk is an important consideration when devising treatment and management plans [21]. Lang et al. assessed the benefits of using an AI 3D angiography technique based on a single contrast-enhanced run that functioned on half the radiation of conventional methods [22]. Similarly, Bernard et al. used DL reconstruction for cardiac CT angiography in an acute stroke imaging protocol and found that the image quality improved, with a significant reduction in radiation compared to iterative reconstruction [23]. This is another example of AI integration in medical practice that makes neurosurgery procedures more sustainable in the long term while improving the quality and accuracy of imaging and reducing patient risk.

Through DL and AI, the healthcare system, in general, can enhance patient treatment and obtain better overall outcomes. Radiomics allows using a large amount of data from radiological images and obtains quantitative characteristics of tumor histology and biomarkers in a non-invasive way, even managing to predict the molecular profile and prognosis and providing more personalized therapy [20]. Other advances in AI promise the integration of DL algorithms of brain MRI scans with robotic surgery. Conventional surgery, including robot-guided surgery, has limitations, such as inaccuracy in the extension of the lesion. AI can provide algorithms that reduce this limitation, strictly delimit the size and location of the lesion, and guide the surgeon in real-time. The surgery is minimally invasive and has fewer complications. Zeineldin et al. determined that the multimodality AI-driven system received a score of 75 out of 100 on the System Usability Scale questionnaire from neurosurgeons and had positive feedback in surgeries [24].

Challenges and limitations of deep learning in the field of brain oncology

One of the most pertinent reasons AI is being sought after in the medical field is the vast amount of data that exists. Management of this data is complicated, time-consuming, and labor-consuming. However, it is essential to understand that although AI is substantially more efficient at processing data, it faces many of the same challenges. A common challenge in all medical research is ensuring that patient privacy is maintained and ethical policies are adhered to, which places stringent restrictions on accessing data [25]. This is especially limiting in DL models as they require a vast amount of data to train their algorithms to a standard of accuracy that is safe for integration into medical interventions [26]. Another essential factor to consider is the data quality, as AI can only be as good as the data used to train it [27]. This barrier impedes the development of accurate CNN models as medical records and data are usually complex, and unconventional presentations occur often.

DL models use processing layers that result in deep CNNs. They include different layers for convolution, pooling, and classification. Data supplied by manual segmentation is used as the standard segmentation criterion. The DL model enables the automatic diagnosis and classification of MRI images, such as Bilsky MESCC, using CNN that evaluates metastatic epidural compression. It employs axial T2-weighted images to provide immediate results and decisions for initial surgical decompression or radiotherapy, with sensitivity and specificity similar to that of a musculoskeletal radiologist, neuroradiologist, and radiation oncologist [28].

Despite the high number of false positives, DL allows for approximately 80% sensitivity in detecting brain metastases. Deep Medic, a 3D CNN, is a DL model architecture operating on MRI data in different centers in T1, T2, contrast-enhanced T1, and fluid-attenuated inversion recovery. The effectiveness of this DL model has also been evaluated in brain metastases from malignant melanoma, with a sensitivity of 88%, with false-positive results and segmentation <1 [29]. Q-learning, associated with a surgical route planning algorithm, is useful for determining optimal entry points into the skull and the most advantageous routes for minimally invasive procedures and tumor removal [30].

It is known that AI ethics guidelines such as transparency, justice, fairness, non-maleficence, responsibility, and autonomy should be followed [31]. However, there is little literature on ethical issues with AI in surgery. In areas where sufficient information is available, the Delphi technique is used. There is a lack of infrastructure for data acquisition and guidelines on data ownership. Not all data are available digitally, and two or more systems cannot exchange data. There is no current consensus for data exchange, which is necessary for the competitiveness of AI, and limitations could arise in the exchange of data across international borders. It was agreed that digital surgery consent procedures should disclose the extent of data collection and its reasons and allow for future data collection. Most legislation in the United States is governed by the Privacy Rule of the Health Insurance Portability and Accountability Act.

There are still many ethical challenges associated with AI and neuro-interventional procedures in general, including algorithmic bias, which results from the decision-making process of the algorithms [32]. Another important ethical consideration is access disparity, as the technology needed to utilize these techniques is expensive. Furthermore, there are many other ethical issues regarding legal responsibilities, data privacy, and licensing that have still not been globally agreed upon [33]. A systematic review assessing the application of ChatGPT in neurosurgical practice and education found that 62% of studies reported ethical considerations as the most significant limitation. Additionally, 15% of studies highlighted question format limitations, validation challenges, algorithmic bias, and potential biases as notable concerns [34].

Future directions and potential impact

One of the most significant factors that has slowed the integration of AI into the medical field is the fear of AI completely taking over and replacing medical practitioners. It is important to remember that AI will never replace humans. Although it may come close to the accuracy that a skilled doctor can achieve, irrefutable evidence demonstrates that combining clinicians and AI yields the best results [35,36]. However, in neurosurgery, AI is generally welcomed, which suggests that the development will be exponential with better and faster integration into neurological procedures [37].

AI has recently been integrated into clinical practice in combination with a brain-computer interface to restore motor and sensory functions in paralysis patients. In these patients, the cortex still generates neural information for motor function, but due to the spinal injury, it does not reach the intended limb muscle. Electroencephalography, a noninvasive method of reading neuronal activity in the cortex, can process the information and link it to robotic arms that can perform the intended movement [38]. This technique, called “neural bypass,” is only one demonstration of how AI can be integrated into clinical practice [39].

It is essential to mention that AI will be a crucial tool in the development of medicine and the more personalized management of conditions. AI can assist clinicians in devising treatment plans based on individual patient characteristics and tumor behavior. AI can analyze the vast amount of data and information on a particular patient, including past medical history, genetic information, and patient preferences. This will result in more personalized treatment plans, which are more efficient and more customed to the patient’s needs [40]. Furthermore, AI can be advanced to understand the limitations of hospitals and resources, leading to better patient population management.

Collaboration between engineers and clinicians will bring about a new era of medicine. Combining AI and neurosurgeons will increase the efficiency and accuracy of procedures and reduce the risk posed by surgery. Although AI has limitations, they can be overcome with careful data monitoring, management of coding bugs, and constant algorithm development [41]. With the oversight of engineers ensuring that the AI is functional and customed to be integrated into clinical practice, neurosurgeons will be more willing to use it as a tool to better healthcare and devise personalized treatment plans.

## Conclusions

Integrating AI into neurosurgery is the next step in a new era of medicine that allows for more personalized patient treatment and management, leading to better patient outcomes. AI has demonstrated in countless experiments and innovations to be more accurate and efficient than conventional methods while also reducing risk to patients via reduced radiation and operation duration. However, there are still many challenges and barriers until AI can be wholly integrated and accepted into neurosurgical procedures. There is a consensus among medical professionals that DL and AI are unequivocally valuable tools for neuro-interventional and surgical procedures. Therefore, further research and time should be dedicated to advancing this field.

## References

[1] Kheirollahi M, Dashti S, Khalaj Z, Nazemroaia F, Mahzouni P (2015). Brain tumors: special characters for research and banking. Adv Biomed Res.

[2] Liu Y, Wu M (2023). Deep learning in precision medicine and focus on glioma. Bioeng Transl Med.

[3] National Research Council (US) Committee on A Framework for Developing a New Taxonomy of Disease (2011). Toward Precision Medicine: Building a Knowledge Network for Biomedical Research and a New Taxonomy of Disease. https://pubmed.ncbi.nlm.nih.gov/22536618/.

[4] Russell S, Norvig P (2010). Artificial Intelligence: A Modern Approach, Third Edition. ResearchGate.

[5] Brody H (2013). Medical imaging. Nature.

[6] Shen D, Wu G, Suk HI (2017). Deep learning in medical image analysis. Annu Rev Biomed Eng.

[7] Hinton GE, Salakhutdinov RR (2006). Reducing the dimensionality of data with neural networks. Science.

[8] LeCun Y, Bengio Y, Hinton G (2015). Deep learning. Nature.

[9] ZainEldin H, Gamel SA, El-Kenawy EM, Alharbi AH, Khafaga DS, Ibrahim A, Talaat FM (2022). Brain tumor detection and classification using deep learning and sine-cosine fitness grey wolf optimization. Bioengineering (Basel).

[10] Kabir Anaraki A, Ayati M, Kazemi F (2019). Magnetic resonance imaging-based brain tumor grades classification and grading via convolutional neural networks and genetic algorithms. Biocybern Biomed Eng.

[11] Abdusalomov AB, Mukhiddinov M, Whangbo TK (2023). Brain tumor detection based on deep learning approaches and magnetic resonance imaging. Cancers (Basel).

[12] Yahyaoui H, Ghazouani F, Farah IR (2024). Deep learning guided by an ontology for medical images classification using a multimodal fusion. 2021 International Congress of Advanced Technology and Engineering (ICOTEN).

[13] Hollon TC, Pandian B, Adapa AR (2020). Near real-time intraoperative brain tumor diagnosis using stimulated Raman histology and deep neural networks. Nat Med.

[14] Novis DA, Zarbo RJ (1997). Interinstitutional comparison of frozen section turnaround time. A College of American Pathologists Q-Probes study of 32868 frozen sections in 700 hospitals. Arch Pathol Lab Med.

[15] Roberts DW, Strohbehn JW, Hatch JF, Murray W, Kettenberger H (1986). A frameless stereotaxic integration of computerized tomographic imaging and the operating microscope. J Neurosurg.

[16] Jolesz FA, Kikinis R, Talos IF (2001). Neuronavigation in interventional MR imaging. Frameless stereotaxy. Neuroimaging Clin N Am.

[17] Tangsrivimol JA, Schonfeld E, Zhang M (2023). Artificial intelligence in neurosurgery: a state-of-the-art review from past to future. Diagnostics (Basel).

[18] Ibrahim M, Muhammad Q, Zamarud A, Eiman H, Fazal F (2023). Navigating glioblastoma diagnosis and care: transformative pathway of artificial intelligence in integrative oncology. Cureus.

[19] Cè M, Irmici G, Foschini C (2023). Artificial intelligence in brain tumor imaging: a step toward personalized medicine. Curr Oncol.

[20] Forghani R (2020). Precision digital oncology: emerging role of radiomics-based biomarkers and artificial intelligence for advanced imaging and characterization of brain tumors. Radiol Imaging Cancer.

[21] Kamiya K, Ozasa K, Akiba S (2015). Long-term effects of radiation exposure on health. Lancet.

[22] Lang S, Hoelter P, Schmidt M (2020). Evaluation of an artificial intelligence-based 3D-angiography for visualization of cerebral vasculature. Clin Neuroradiol.

[23] Bernard A, Comby PO, Lemogne B, Haioun K, Ricolfi F, Chevallier O, Loffroy R (2021). Deep learning reconstruction versus iterative reconstruction for cardiac CT angiography in a stroke imaging protocol: reduced radiation dose and improved image quality. Quant Imaging Med Surg.

[24] Zeineldin RA, Junger D, Mathis-Ullrich F, Burgert O (2024). Development of an AI-driven system for neurosurgery with a usability study: a step towards minimal invasive robotics. Automatisierungstechnik.

[25] Deo RC (2015). Machine learning in medicine. Circulation.

[26] Senders JT, Staples PC, Karhade AV (2018). Machine learning and neurosurgical outcome prediction: a systematic review. World Neurosurg.

[27] Mofatteh M (2021). Neurosurgery and artificial intelligence. AIMS Neurosci.

[28] Awuah WA, Adebusoye FT, Wellington J (2024). Recent outcomes and challenges of artificial intelligence, machine learning, and deep learning in neurosurgery. World Neurosurg X.

[29] Pennig L, Shahzad R, Caldeira L (2021). Automated detection and segmentation of brain metastases in malignant melanoma: evaluation of a dedicated deep learning model. AJNR Am J Neuroradiol.

[30] Dundar TT, Yurtsever I, Pehlivanoglu MK (2022). Machine learning-based surgical planning for neurosurgery: artificial intelligent approaches to the cranium. Front Surg.

[31] Lam K, Abràmoff MD, Balibrea JM (2022). A Delphi consensus statement for digital surgery. NPJ Digit Med.

[32] Wang C, Liu S, Yang H, Guo J, Wu Y, Liu J (2023). Ethical considerations of using ChatGPT in health care. J Med Internet Res.

[33] Guleria A, Krishan K, Sharma V, Kanchan T (2024). ChatGPT: forensic, legal, and ethical issues. Med Sci Law.

[34] Bečulić H, Begagić E, Skomorac R, Mašović A, Selimović E, Pojskić M (2024). ChatGPT's contributions to the evolution of neurosurgical practice and education: a systematic review of benefits, concerns and limitations. Med Glas (Zenica).

[35] Yamashita K, Yoshiura T, Arimura H (2008). Performance evaluation of radiologists with artificial neural network for differential diagnosis of intra-axial cerebral tumors on MR images. AJNR Am J Neuroradiol.

[36] Kitajima M, Hirai T, Katsuragawa S (2009). Differentiation of common large sellar-suprasellar masses effect of artificial neural network on radiologists' diagnosis performance. Acad Radiol.

[37] Layard Horsfall H, Palmisciano P, Khan DZ, Muirhead W, Koh CH, Stoyanov D, Marcus HJ (2021). Attitudes of the surgical team toward artificial intelligence in neurosurgery: international 2-stage cross-sectional survey. World Neurosurg.

[38] Zhang X, Ma Z, Zheng H (2020). The combination of brain-computer interfaces and artificial intelligence: applications and challenges. Ann Transl Med.

[39] Bouton CE, Shaikhouni A, Annetta NV (2016). Restoring cortical control of functional movement in a human with quadriplegia. Nature.

[40] Sümerkent K (2023). AI and neurosurgery: exploring the future of brain surgery. Medium.

[41] Iqbal J, Jahangir K, Mashkoor Y (2022). The future of artificial intelligence in neurosurgery: a narrative review. Surg Neurol Int.

